# Interaction Mechanisms Between the NOX4/ROS and RhoA/ROCK1 Signaling Pathways as New Anti- fibrosis Targets of Ursolic Acid in Hepatic Stellate Cells

**DOI:** 10.3389/fphar.2019.00431

**Published:** 2019-05-03

**Authors:** Chenkai Huang, Dakai Gan, Fangyun Luo, Sizhe Wan, Jiang Chen, Anjiang Wang, Bimin Li, Xuan Zhu

**Affiliations:** ^1^Department of Gastroenterology, The First Affiliated Hospital of Nanchang University, Nanchang, China; ^2^Department One of Liver Disease, The Ninth Hospital of Nanchang, Nanchang, China; ^3^Digestive Disease Research Institute of Jiangxi Province, Nanchang, China

**Keywords:** hepatic stellate cells, NOX4, RhoA, cytoskeleton, ursolic acid

## Abstract

**Background:**

Studies have shown that both NOX4 and RhoA play essential roles in fibrosis and that they regulate each other. In lung fibrosis, NOX4/ROS is located upstream of the RhoA/ROCK1 signaling pathway, and the two molecules are oppositely located in renal fibrosis. Currently, no reports have indicated whether the above mechanisms or other regulatory mechanisms exist in liver fibrosis.

**Objectives:**

To investigate the effects of the NOX4/ROS and RhoA/ROCK1 signaling pathways on hepatic stellate cell (HSC)-T6 cells, the interaction mechanisms of the two pathways, and the impact of UA on the two pathways to elucidate the role of UA in the reduction of hepatic fibrosis and potential mechanisms of HSC-T6 cell proliferation, migration, and activation.

**Methods:**

Stable cell lines were constructed using the lentiviral transduction technique. Cell proliferation, apoptosis, migration, and invasion were examined using the MTS, TdT-mediated dUTP nick-end labeling, cell scratch, and Transwell invasion assays, respectively. The DCFH-DA method was used to investigate the ROS levels in each group. RT-qPCR and western blotting techniques were utilized to assess the mRNA and protein expression in each group. CoIP and the Biacore protein interaction analysis systems were used to evaluate protein interactions.

**Results:**

The NOX4/ROS and RhoA/ROCK1 signaling pathways promoted the proliferation, migration, and activation of HSCs. UA inhibited cell proliferation, migration, and activation by inhibiting the activation of the two signaling pathways, but the mechanism of apoptosis was independent of these two pathways. The NOX4/ROS pathway was upstream of and positively regulated the RhoA/ROCK1 pathway in HSCs. No direct interaction between the NOX4 and RhoA proteins was detected.

**Conclusion:**

The NOX4/ROS and RhoA/ROCK1 signaling pathways are two critical signaling pathways in a series of behavioral processes in HSCs, and NOX4/ROS regulates RhoA/ROCK1 through an indirect pathway to control the activation of HSCs. Additionally, NOX4/ROS and RhoA/ROCK1 constitute a new target for UA antifibrosis treatment.

## Introduction

Hepatic fibrosis is the process by which a variety of etiologies cause the proliferation and transformation of HSCs into MFBs, leading to extracellular matrix (ECM) deposition or scar formation (Schuppan et al., 2018). The sustained development of liver fibrosis can eventually evolve into cirrhosis and even liver cancer, which seriously endangers human health (Lee et al., 2015). The transformation of HSCs into proliferating MFBs is a central event in the pathogenesis of hepatic fibrosis. Activated HSCs/MFBs cause a series of changes in liver metabolism (Shang et al., 2018). Therefore, inhibits the activation of HSC and activates senescence and apoptosis of HSC are the keys to reversing hepatic fibrosis (Tacke, 2017).

Nicotinamide adenine dinucleotide phosphate oxidase (NOX) is a multisubunit transmembrane protein complex whose primary function is to use NADPH to transfer electrons to O_2_ molecules to produce $O_{2}^{–}$ and H_2_O_2_ (Crosas-Molist and Fabregat, 2015). The NOX family participates in the regulation of signal transduction in HSCs by generating ROS and plays a vital role in the activation of HSCs and the pathogenesis of hepatic fibrosis (Paik et al., 2011). The activity of NOX4 is mainly regulated by p22^phox^ and Poldip2 (Sirokmany et al., 2016). Aoyama et al. (2012) showed that both TGF-β1 and Ang II upregulate NOX4 expression and that a dual inhibitor of NOX1/4, GKT137831, inhibits ROS production and hepatic fibrosis. These findings indicate that NOX4 mediates the signal transduction of TGF-β1 and other major hepatic fibrogenic factors in HSCs, leading to their activation. Thus, NOX4 plays an essential role in the development of hepatic fibrosis.

More than 20 members of the Rho GTPase superfamily have been identified, and RhoA is one of the most studied Rho GTPases (Nakamura et al., 2017) and is involved in a variety of cellular activities. Studies have shown that RhoA and its downstream signaling molecules are expressed in hepatic vascular smooth muscle cells, vascular endothelial cells, and HSCs, increasing hepatic vascular resistance and aggravating hepatic fibrosis (Nomikou et al., 2018). Recent studies have found that RhoA regulates liver fibrosis by controlling HSC activity. First, RhoA activates HSCs and synthesizes α-SMA, a major component of the cytoskeleton and an activated HSC/MFB marker. Second, RhoA acts on MFBs; changes the cytoskeleton (Ni et al., 2013); and regulates the migration, adhesion, and contraction of HSCs, thereby accelerating their activation (Li et al., 2012; Klein et al., 2017). Thus, RhoA participates in the regulation of hepatic fibrosis by regulating the activation, migration, adhesion, contraction, and proliferation of HSCs.

The relationship between RhoA/ROCK and NOX4/ROS remains controversial. Meng et al. (2015) reported that NOX4/ROS activates the RhoA/ROCK1 signaling pathway, promotes lung fibroblast migration, promotes collagen synthesis, and increases pulmonary fibrosis. Interestingly, RhoA/ROCK1 is a signaling pathway upstream of NOX4/ROS that promotes the differentiation of renal muscle fibroblasts and aggravates renal fibrosis (Manickam et al., 2014). Although both RhoA/ROCK1 and NOX4/ROS are involved in the regulation of cell activation and fibrosis (Paik et al., 2014), the mutual regulation of RhoA/ROCK1 and NOX4/ROS in hepatic fibrosis has not been reported.

Our previous studies have confirmed that UA inhibits the NOX production of ROS in HSCs and that NOX4/ROS is a target of antifibrotic UA. Rac1 is involved in regulating the activation of NOX subunits and HSCs, and UA inhibits the expression of Rac1, a Rho GTPase family member (Yu et al., 2017). In addition, UA inhibits activation of the HSC fibrotic signaling network, as it inhibits the NOX, Rac1, NF-κB, PI3K/Akt, P38MAPK, ERK1/2, JAK2-STAT3, and Hedgehog signaling pathways (He et al., 2015; Gan et al., 2018). The present study investigated the interaction between NOX4/ROS and RhoA/ROCK1 in hepatic fibrosis, the direct binding of NOX4 and RhoA, and the specific antifibrosis molecular targets of UA.

## Materials and Methods

### Reagents

Transforming growth factor-β1 (cat #: T1654), UA (cat #: 03240595), and protein A agarose (cat #: P1406) were purchased from Sigma–Aldrich (St. Louis, MO, United States). Matrigel (cat #: 354263) was purchased from Corning Incorporated (New York, NY, United States). Puromycin (cat #: REVG1001) was purchased from GENECHEM (Shanghai, China). The anti-NOX4 (cat #: ab109225), anti-RhoA (cat #: ab187027), anti-ROCK1 (cat #: ab205829), anti-α-SMA (cat #: ab32575), anti-Collagen-I (cat #: ab6308), anti-p67^phox^ (cat #: ab109366), anti-Rac1 (cat #: ab33186), anti-TIMP1 (cat #: ab61224), anti-MMP1 (cat #: ab137332), anti-MMP2 (cat #: ab37150), anti-MMP9 (cat #: 119906), and anti-F-actin (cat #: ab205) antibodies were purchased from Abcam (Cambridge, MA, United States). The anti-GAPDH antibody was purchased from OriGene (cat #: TA802519) (Rockville, MD, United States). The full-length human recombinant NOX4 (cat #: H00050507-G01) and RhoA (cat #: H00000387-P01) proteins were purchased from Abnova (Walnut, CA, United States).

### Mice

Wild-type (WT) C57BL/6 mice were obtained from the Department of Laboratory Animal Science of Nanchang University, and N_OX_4^-/-^ mice were obtained from The Jackson Laboratory (Bar Harbor, ME, United States). Animals were maintained in an environment with a 12:12 h light/dark cycle, a room temperature of 22 ± 2°C, and a 55 ± 5% humidity. Male WT and NOX4^-/-^ mice were randomly divided into the following four groups (*n* = 10/group): control, CCl_4_ model, UA treatment, and Fasudil treatment; the control mice were administered olive oil (2 ml/kg) by gavage twice a week for 8 weeks and then administered normal saline (40 mg/kg/d) for 4 weeks. The early stage of hepatic fibrosis was induced by gastric gavage administration of CCl_4_ (diluted 1:4 in olive oil, 2 ml/kg) twice a week for 8 weeks, after which the mice in the CCl_4_ model and UA/Fasudil treatment groups were given normal saline or UA (40 mg/kg/d)/Fasudil (10 mg/kg/bid) for 4 weeks, respectively. All experimental procedures were approved by the Institutional Animal Care and Use Committee of the First Affiliated Hospital of Nanchang University (Nanchang, China). All animals received humane care in compliance with institutional guidelines. Liver samples were harvested for hematoxylin and eosin (HE) & Masson staining.

### HE and Masson Staining

Paraffin-embedded liver samples were cut into 5-μm-thick slices with a microtome and then stained with HE using standard methods. For Masson staining, samples were deparaffinized and rehydrated through a series of 100% alcohol, 95% alcohol, and 70% alcohol, washed in distilled water, and then rinsed under running tap water for 5–10 min to remove the yellow color. The samples were then stained in Weigert’s iron hematoxylin working solution for 10 min, rinsed under running warm tap water for 10 min, and washed in distilled water. Next, the samples were stained with Biebrich scarlet-acid fuchsin solution for 10–15 min, washed in distilled water, and differentiated in a phosphomolybdic–phosphotungstic acid solution for 10–15 min or until collagen was not red. The sections were directly (without rinsing) transferred to an aniline blue solution and stained for 5–10 min before being rinsed briefly in distilled water and differentiated in a 1% acetic acid solution for 2–5 min. Finally, the sections were washed in distilled water, quickly dehydrated via 95% ethyl alcohol and absolute ethyl alcohol, and cleared in xylene.

### HSC-T6 Cell Culture and Stimulation

The HSC-T6 cell line was purchased from the Type Culture Collection of the Chinese Academy of Sciences in Shanghai, China and cultured in Dulbecco’s Modified Eagle’s Medium (DMEM) (HyClone, South Logan, UT, United States) supplemented with 10% fetal bovine serum (FBS, Gibco, Grand Island, NY, United States) at 37°C and 5% CO_2_. Upon reaching 80–90% confluence, cells were grown in serum-free medium for 24 h before being used in experiments. For the experiments, HSCs were pretreated with DMEM containing UA (50 μmol/L, 0.1% DMSO) for 30 min and then treated with DMEM containing TGF-β1 (10 ng/mL). The culture continued for 48 h.

### Lentivirus Transduction

All packaged lentiviruses were obtained from GENECHEM (Shanghai, China). The knockdown oligonucleotide sequences were as follows: scramble, 5′-GUAAGACACGACUUAUCGC-3′; Rat NOX4, 5′-TCCCTCAGATGTCATGGAA-3′; and Rat RhoA, 5′-CCCAGACACTGATGTTATA-3′. Specific oligonucleotides with maximal knockdown efficiency were selected from three different sequences for each gene. The NOX4 overexpression lentivirus was generated using the GV358 vector and AgeI/AgeI digestion. The overexpression primer sequences were as follows: 5′-GAGGATCCCCGGGTACCGGTCGCCACCATGGCGCTGTCCTGGAGGAGCTGG-3′ and 5′-TCCTTGTAGTCCATACCGCTGAAAGATTCTTTATTGTATTCAAATTTTG-3′. A multiplicity of infection (MOI) gradient experiment was performed to identify the most suitable MOI value for the HSC-T6 cell line, and this value was used for the transduction experiments. In brief, blank HSC-T6 cells were seeded into 24-well culture plates at 1–5 × 10^4^ cells per well (approximately equal to 20–35% confluency). After diluting puromycin with cell culture medium (1000-fold for a final concentration of 1 μg/mL), the culture medium with puromycin was added to each well of the 24-well culture plate, and the plate was placed in an incubator at 37°C and 5% CO_2_. Cells were incubated overnight and observed using a fluorescence microscope every 12 h for 3 days. Surviving cells were used for the next experiments, and none of the stable cells were passaged more than seven times.

### Cell Viability

Cell viability was measured by the MTS kit (Promega, Madison, WI, United States). In brief, 1000 cells per well were seeded into a 96-well plate, and UA was added according to the group requirements. MTS solution (20 μL) was added to each well, and cells were incubated for 4 h. The absorbance of each well was measured at 490 nm using a microplate reader.

### Apoptosis Detection

TdT-mediated dUTP nick-end labeling Apoptosis Assay Kit (Abcam, Cambridge, MA, United States) was used to detect the apoptosis of each group. Cells were washed with PBS, resuspended, inoculated on a slide, placed on a clean bench for 15 min, and washed twice with PBS. Cells were then fixed with 4% paraformaldehyde for 25 min and washed twice with PBS for 5 min. Cells were then permeabilized with 0.2% Triton X-100 for 5 min and washed twice with PBS for 5 min. Excess liquid was removed from the filter paper, and 100 μL of equilibration buffer was added. The slide was covered and incubated for 10 min at room temperature. The slide system was placed on ice, and excess liquid was aspirated from the filter paper. A TdT enzyme reaction solution (100 μL/well) was added to prevent drying. The reaction system was placed in a wet box and incubated at 37°C for 1 h. Then, 2× SSC was added dropwise, and slides were incubated for an additional 15 min. Slides were washed three times with PBS for 5 min, and 0.3% hydrogen peroxide (diluted with PBS) was added for 5 min followed by three washes with PBS for 5 min. A streptavidin horseradish peroxidase (HRP) working solution (100 μL) was then added and allowed to react for 30 min, and the slides were then washed three times with PBS for 5 min. A DAB working solution (100 μL) was added until a brown background appeared, and the slides were then washed several times with double distilled water. The slides were mounted, and staining was observed using a microscope.

### Cell Scratch Test

Before the test, horizontal lines (every 1 cm across the hole and 5 lines per hole) were drawn on the back of a 6-well plate with a marker. Approximately 10^5^ cells were added to the wells and treated for approximately 48 h. After the treatment, a pipette tip was used to scratch the bottom of the cell culture perpendicular to the horizontal back line. Cells were washed three times with PBS, and serum-free DMEM was added. Cells were placed in an incubator to continue culturing, and images were acquired at 12 h.

### Transwell Invasion Assay

The upper surface of the bottom membrane of the Transwell chamber was coated with a 50 mg/L Matrigel 1:8 solution and air-dried at 4°C. Then, 10% complete medium was added to the culture plate, and the chamber was placed in the culture plate to avoid the formation of air bubbles between the bottom film and the culture medium. The cell density was adjusted to 10^5^ in the chamber. Each group was treated accordingly and incubated for 48 h. Cells were evaluated using the MTS indirect counting method. Matrigel and cells were removed from the chambers. Complete medium (500 μL) containing 0.5 mg/mL MTS was added to each well, and the chambers were placed back in the wells. Membranes were immersed in culture medium and incubated at 37°C for 4 h. The culture medium was then removed, and 500 μL of DMSO was added. The plate was shaken for 10 min, and the chambers were then removed. The optical density (OD) value was then determined using a microplate reader.

### Determination of ROS

After cell stimulation, a single cell suspension of HSCs was made, and cells were uniformly seeded into 6-well plates at approximately 4 × 10^5^ cells per well. Cells were cultured overnight in humidified air containing 5% CO_2_ at 37°C and then digested with 0.25% trypsin without EDTA. After termination of digestion, cells were centrifuged at 1,500 rpm for 5 min. Supernatants were discarded, and pellets were resuspended in PBS. The above steps were repeated three times. DCFH-DA was used to detect ROS by adding 1 mL of PBS containing diluted DCFH to the cells. The cells were incubated at 37°C for 20 min and mixed every 3 min during this period. The cells were then washed three times with serum-free medium and resuspended in 500 μL of PBS. A flow cytometer was used to detect cells at excitation and emission wavelengths of 525 and 485 nm, respectively. The results are expressed as fluorescence intensity/mg protein using the following equation: ROS (fluorescence intensity/mg protein) = fluorescence intensity/(protein concentration × 0.19).

### RNA Preparation and RT-qPCR

Total RNA was prepared from frozen tissue samples or cells using the RNA Simple Total RNA Kit and the Fast Quant RT Kit (Tiangen, Beijing, China). The concentration and purity of isolated RNA were determined by measuring the OD at 260 and 280 nm. The integrity of the RNA was verified using agarose gel electrophoresis. For RT-qPCR, the Fast Quant RT Kit, and Super Real Pre-Mix Plus (SYBR Green, Tiangen, Beijing, China) were used according to the manufacturer’s protocol. The primer sets are shown in Table 1. All primers were obtained from Beijing Genomics Institute (BGI, Shenzhen, China).

**Table 1:** Primer sequences for RT-qPCR.

		Amplicon
Gene	Sequence	length (bp)
*Nox4*	F: 5′-CCTCAGTCAAACAGATGGGATA-3′	169
	R: 5′-GGAAATAGAACTGGGTCCACA-3′	
*Rhoa*	F: 5′-GCAGGTAGAGTTGGCTTTATGG-3′	231
	R: 5′-CTTGTGTGCTCATCATTCCGA-3′	
*Rock1*	F: 5′-TGGAAAGACATGCTTGCTCAT-3′	133
	R: 5′-CGGTTAGAACAAGAGGTAAAT-3′	
*Alphasma*	F: 5′-CGATAGAACACGGCATCATC-3′	525
	R: 5′-CATCAGGCAGTTCGTAGCTC-3′	
*Col1a1*	F: 5′-GGGGCAAGACAGTCATCGAA-3′	144
	R: 5′-GGATGGAGGGAGTTTACACGAA-3′	
*Mmp1*	F: 5′-GCTGATACTGACACTGGTACTG-3′	216
	R: 5′-CAATCTTTTCTGGGAGCTC-3′	
*Timp1*	F: 5′-CCACAGATATCCGGTTCGGCTACA-3′	218
	R: 5′-GCACACCCCACAGCCAGCACTAT-3′	
*Gapdh*	F: 5′-TTCAACGGCACAGTCAAGG-3′	114
	R: 5′-CTCAGCACCAGCATCACC-3′	

### Western Blot Analysis

Protein was isolated from cells by lysis (BestBio, Shanghai, China) with phenylmethanesulfonyl fluoride (PMSF, cat #: 8553, Cell Signaling Technology, Danvers, MA, United States) for western blotting. Cells were harvested and resuspended in PBS, and cell protein extracts were obtained by centrifugation at 12,000 rpm for 10 min. Protein levels were determined using the BCA assay kit (Tiangen, Beijing, China). Protein extracts (10–20 μL) were separated using 6–12% sodium dodecyl sulfate polyacrylamide gel electrophoresis and electrophoretically transferred onto polyvinylidene fluoride membranes. After blocking with 5% nonfat dried milk in Tris-buffered saline, the PVDF membranes were incubated overnight at 4°C with the rabbit anti-NOX4 (1:1000), anti-RhoA (1:1000), anti-ROCK1 (1:1000), anti-α-SMA (1:200), anti-collagen-I (1:1000), anti-MMP1 (1:2000), anti-TIMP1 (1:2000), anti-F-actin (1:2000), and anti-GAPDH (1:1000) antibodies. A secondary HRP-conjugated anti-rabbit or anti-mouse IgG antibody (Zhongshan Golden Bridge Biotechnology Co., Beijing, China) was then added, and specific bands were visualized using an enhanced chemiluminescence (ECL) detection kit (Thermo Fisher Scientific Inc., Waltham, MA, United States).

### Immunofluorescence Cytochemistry

Cells were fixed in chilled methanol (-30°C) on ice for 5 min. After incubation in a blocking solution (10% goat serum, Grand Island, New York, NY, United States) at 4°C for 30 min, cells were incubated with primary antibodies at room temperature for 1 h or at 4°C overnight. Then, the primary antibodies were detected using DyLight 594-conjugated secondary antibodies (cat #: 12877, Cell Signaling Technology, Danvers, MA, United States). Nuclei were counterstained with DAPI. Finally, the images were acquired under a fluorescence microscope.

### Coimmunoprecipitation

An appropriate amount of lysate was added to the cell culture plate, which was placed on ice for 30 min for full lysis. The lysates were centrifuged at 12,000 rpm for 30 min, and the supernatant was collected. A small amount of lysate was used for subsequent WB analysis. The appropriate corresponding antibody (1 μg) was added to the remaining lysate, which was gently shaken and incubated overnight at 4°C. Protein A agarose beads (10 μL) were washed three times with an appropriate amount of lysis buffer and centrifuged at 3000 rpm for 3 min. Pretreated protein A agarose beads (10 μL) were added to the cell lysate, incubated for 4 h at 4°C with gentle shaking and centrifuged at 3000 rpm for 3 min at 4°C. The supernatant was removed with a pipette, and the agarose beads were washed three times with 1 mL of lysis buffer. Then, 15 μL of 2× SDS loading buffer was added, and samples were incubated in a 100°C metal bath for 5 min and subjected to WB analysis.

### Biacore Protein Interaction Analysis

For the prebinding experiment, pure NOX4 protein was diluted 10–40 times with buffers with different pH values and flowed across the surface of the chip at a rate of 5 μL/min. Adsorption of the antibody and chip was observed, and the subsequent CM5 chip coupling was selected by combining the most suitable binding concentration. For CM5 chip coupling, 100 μL of *N*-hydroxysuccinimidobiotin (NHS) was mixed with 100 μL of EDC, and the samples were centrifuged at 12,000 rpm for 5 min. A certain volume of RhoA protein was diluted appropriately according to the preconjugation ratio. Ethanolamine HCl (100 μL) was added to 150 μL of the reserve, equilibrated at room temperature (25°C), and centrifuged at 12,000 rpm for 5 min. The above three reagents were placed on the rack, and the sample flow rate was set to 10 μL/min. The samples were injected using the following volumes: 100, 100, and 60 μL of NHS/EDC, sample, and ethanolamine-HCl, respectively. For conjugation, the purified RhoA protein was diluted to the appropriate concentration (0, 22.5, 45, 90, 180, and 360 μg/mL) with buffer and loaded to observe binding activity. The binding dissociation curve was obtained at different concentrations. For chip regeneration, the chip was flushed with 10 mmol/L glycine (pH 2.5) to restore the baseline to initial values. The results were then analyzed.

### Analysis and Statistical Processing

Image Pro Plus 6.0 software was used for image analysis, and SPSS 23.0 software was used for data analysis. GraphPad Prism 7.0 software was used for image production and output. Each experiment was repeated three times, and data are expressed as the mean ± SD. If the data obeyed the normal distribution and homogeneity of variance, one-way ANOVA was used to analyze the groups of samples. If the data did not follow a normal distribution or variance, a rank sum test was used (Kruskal–Wallis H or Wilcoxon test). *P* < 0.05 was considered statistically significant.

## Results

### UA and Fasudil Attenuate Early-Stage Hepatic Fibrosis Induced by CCl_4_

The initial phase of hepatic fibrosis was established in a mouse model using CCl_4_ for analysis of the antifibrotic effects of UA and Fasudil. As shown in the upper panel of Supplementary Figure 1, after treatments with UA and Fasudil, the collagen deposition in liver tissues was reduced significantly, and the deposition of collagen in the Nox4^-/-^ mouse groups was substantially lower than that in the WT groups regardless of whether UA or Fasudil was used (Supplementary Figure 1 bottom panel).

### NOX4 and RhoA Are Required for the Proliferation of HSCs, and UA Suppresses Their Expression *via* NOX4

**FIGURE 1:**
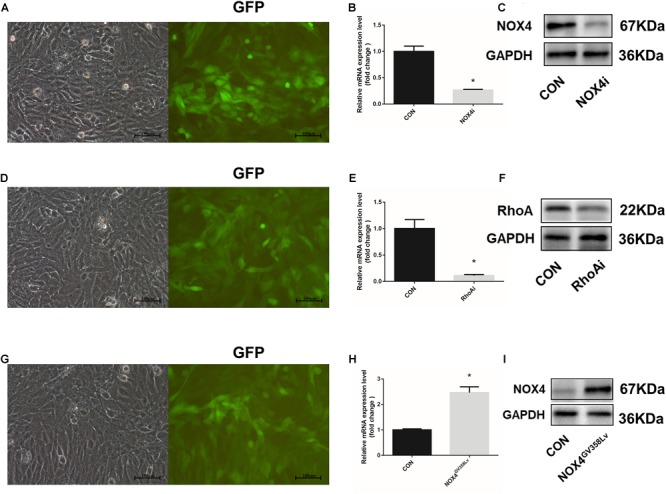
Construction and identification of stable cell lines. **(A)** The HSC-T6 cell line was transfected with a lentivirus containing NOX4 shRNA. Cells were observed under an optical microscope (left) and a fluorescence (GFP) microscope (right) in the same field of vision. **(B)** RT-qPCR analysis of Nox4 mRNA in the NOX4 shRNA stable cell line after TGF-β1 treatment (10 ng/mL) for 48 h. **(C)** WB analysis of the NOX4 protein levels in the NOX4i group. **(D)** The HSC-T6 cell line was transfected with a lentivirus containing RhoA shRNA. Cells were observed under an optical microscope (left) and a fluorescence microscope (right) in the same field of vision. **(E)** RT-qPCR analysis of Rhoa mRNA in the Rhoa shRNA stable cell line. **(F)** WB analysis of the RhoA protein levels in the RhoAi group. **(G)** The HSC-T6 cell line was transfected with a Nox4 overexpression lentivirus. Cells were observed under an optical microscope (left) and a fluorescence microscope (right) in the same field of vision. **(H)** RT-qPCR analysis of Nox4 mRNA in the NOX4 overexpression stable cell line. **(I)** WB analysis of the NOX4 protein levels in the NOX4^GV 358Lv^ group. Original magnification, 200×. The data are presented as the means ± SEMs of three replicates. ^∗^*p* < 0.05 versus the CON group (lentivirus containing a scramble sequence).

Transforming growth factor-β1 (10 ng/mL) was added to the culture medium for 48 h, and the Nox4 mRNA and protein expression levels were compared between the scramble lentivirus control HSCs (CON group) and NOX4 shRNA lentivirus transduction HSCs (NOX4i group). The Nox4 mRNA and protein expression levels in the NOX4i group were significantly decreased compared to those in the control group (*P* < 0.05) (Figure 1B,C). The RhoA shRNA (RhoAi group) and NOX4 overexpression (NOX4^GV 358Lv^ group) cells are shown in Figure 1E,F,H,I. Adherent HSCs were imaged for visual contrast in the same field using light and fluorescence microscopy (Figure 1A,D,G). All groups of cells subjected to lentiviral particles had good regulatory effects and were thus used in subsequent experiments.

**FIGURE 2:**
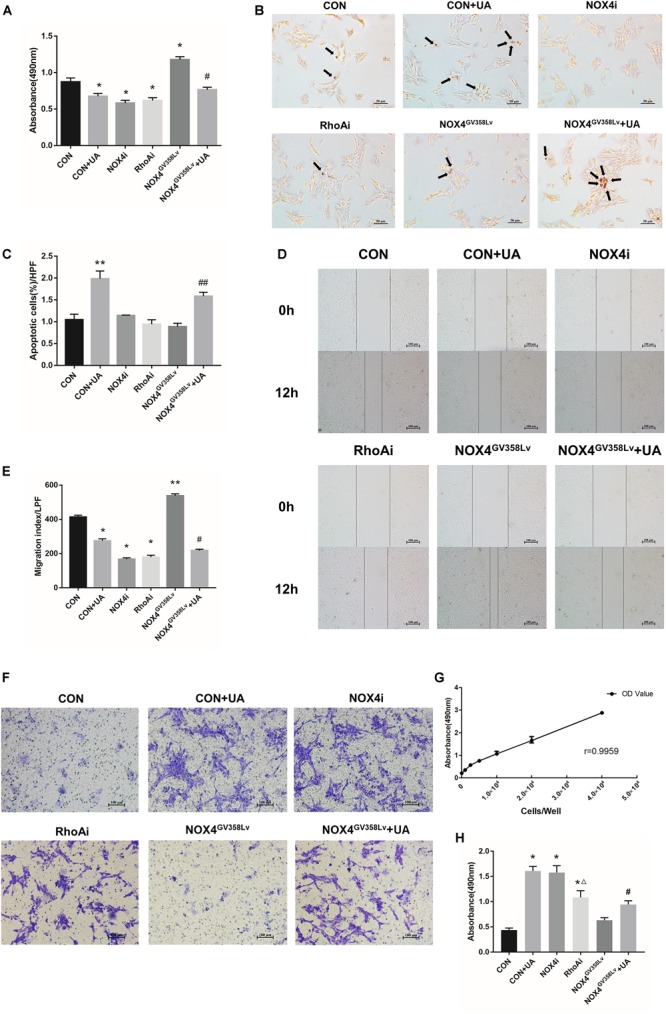
The regulatory effects of NOX4 and RhoA as well as the UA intervention effects on the migration and invasion of HSCs after TGF-β1 treatment (10 ng/mL) for 48 h. **(A)** Cell proliferation detected by the MTS assay. **(B,C)** Cell apoptosis levels detected by the TUNEL assay. Black arrows: typical apoptotic cells. Original magnification, 200×. Percentage of apoptotic cells = apoptotic cells/cell count per high-power field; 10 different fields of vision were randomly selected in each group. **(D,E)** Cell scratch assay. Original magnification, 100×. **(F,H)** Cell invasion detected by the Transwell invasion system. Original magnification, 200×; **(G)** MTS indirect cell count curve. *r* = 0.9959. The data are presented as the means ± SEMs of three replicates. ^∗^*p* < 0.05, ^∗∗^*p* < 0.01 versus the CON group; ^#^*p* < 0.05, ^##^*p* < 0.01 versus the NOX4^GV 358Lv^ group. ∆*p* < 0.05 versus the NOX4i group.

After successful establishment of the cell lines, we further discovered that the proliferation of the CON+UA, NOX4i, and RhoAi groups was lower than that of the CON group (*P* < 0.05). The proliferation of the NOX4^GV 358Lv^ group was elevated compared to that of the CON group (*p* < 0.05), and the proliferation of the NOX4^GV 358Lv^+UA group was lower than that of the NOX4^GV 358Lv^ group (*p* < 0.05) (Figure 2A). These results showed that NOX4 and RhoA promote the proliferation of HSCs and that UA inhibits the proliferation of HSCs *via* UA.

Next, the apoptosis rate of each group was detected (Figure 2B). The proportion of apoptotic cells in the CON+UA group was significantly higher than that in the CON group (*p* < 0.01). The ratios of apoptotic cells in the NOX4i, RhoAi, and NOX4^GV 358Lv^ groups were not significantly different from that in the CON group, and the percentage of apoptotic cells in the NOX4^GV 358Lv^+UA group was considerably higher than that in the NOX4^GV 358Lv^ group (*p* < 0.01) (Figure 2C). Therefore, these results suggested that NOX4 and RhoA do not affect the apoptosis of HSCs, and UA can induce the apoptosis of HSCs independent of either of them.

### NOX4 and RhoA Are Required for the Migration and Invasion of HSCs, and UA Affects Their Expression *via* NOX4

We next aimed to evaluate whether interrupting NOX4 and RhoA expression had any effect on the migration, invasion, or activation of HSCs. The migration indexes of the CON+UA, NOX4i, and RhoAi groups were lower than that of the CON group (*p* < 0.05). The migration index of the NOX4^GV 358Lv^ group was significantly higher than that of the CON group (*p* < 0.01), and the migration index of the NOX4^GV 358Lv^+UA group was lower than that of the NOX4^GV 358Lv^ group (*p* < 0.05) (Figure 2D,E). These results demonstrated that NOX4 and RhoA promote the migration of HSCs and that UA inhibits the migration of HSCs.

The Transwell invasion system was used to assess the levels of HSC invasion in each group (Figure 2F). The standard curve of the cell amounts and the absorbance at 490 nm are shown in Figure 2G. After treatment of HSCs with TGF-β1 (added to Transwell chambers, not to plates), the degrees of invasion of the CON+UA, NOX4i, and RhoAi groups were higher than that of the CON group (*p* < 0.05). The invasion degree of the RhoAi group was lower than that of the NOX4i group (*p* < 0.05), and the invasion degree of the NOX4^GV 358Lv^ group was not significantly different from that of the CON group. The degree of invasion of the NOX4^GV 358Lv^+UA group was increased compared to that of the NOX4^GV 358Lv^ group (*p* < 0.05) (Figure 2H, the standard curve of the MTS cell counts is shown in Figure 2G). These results demonstrated that NOX4 inhibits the invasion of HSCs, RhoA promotes the invasion of HSCs, and UA may promote the invasion of HSCs *via* NOX4.

### NOX4 and RhoA Promote Cytoskeleton F-Actin Polymerization in HSCs, and UA Attenuates Polymerization *via* NOX4

**FIGURE 3:**
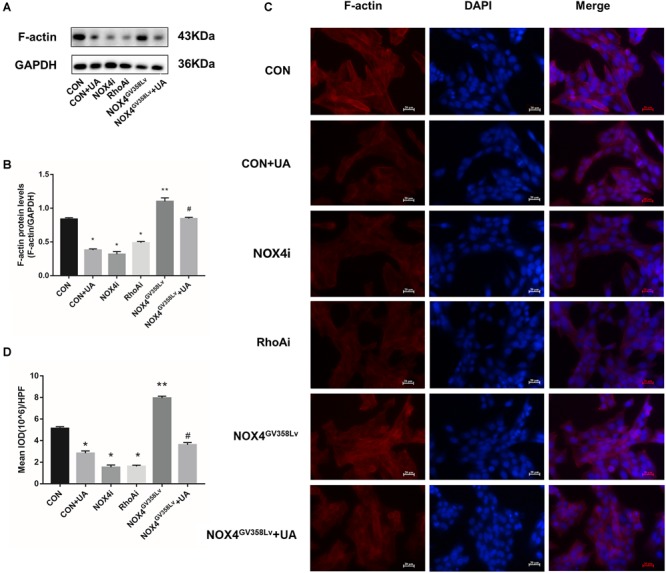
The regulatory effects of NOX4 and RhoA as well as the UA intervention effects on the F-actin polymerization levels in each group after TGF-β1 treatment (10 ng/mL) for 48 h. **(A,B)** WB analysis of F-actin in each group. The data are presented as the means ± SEMs of three replicates. ^∗^*p* < 0.05, ^∗∗^*p* < 0.01 versus the CON group; ^#^*p* < 0.05, ^##^*p* < 0.01 versus the NOX4^GV 358Lv^ group. **(C,D)** F-actin immunofluorescence staining in each group. Mean integral optical density (IOD) values were measured by Image Pro Plus 6.0 software; 10 different fields of vision were randomly selected. Original magnification, 400×.

The protein expression level (Figure 3A,B) and immunofluorescence staining intensity (Figure 3C,D) of F-actin in the CON+UA, NOX4i, and RhoAi groups were lower than those in the CON group (*p* < 0.05), and these values were significantly increased in the NOX4^GV 358Lv^ group compared to those in the CON group (*p* < 0.01). The NOX4^GV 358Lv^+UA group exhibited a lower F-actin staining intensity and protein content than the NOX4^GV 358Lv^ group (*p* < 0.05). These results showed that NOX4 and RhoA promote F-actin polymerization in HSCs and that UA reduces F-actin polymerization in HSCs by suppressing the expression of NOX4.

### UA Reduces the mRNA Expression of the NOX4 and RhoA/ROCK1 Signaling Pathways in HSCs

**FIGURE 4:**
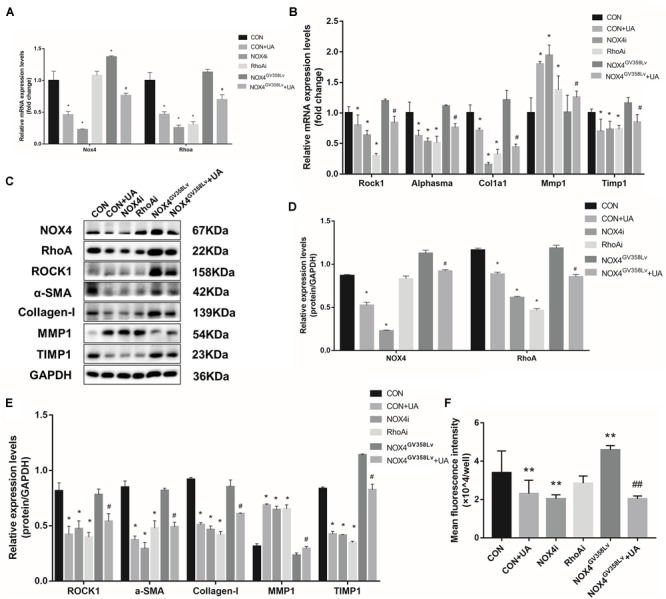
UA intervention effects on the mRNA and protein expression of the NOX4/ROS and RhoA/ROCK1 signaling pathways. **(A,B)** RT-qPCR analysis of Nox4, Rhoa, Rock1, Alphasma, Col1a1, mmp1, and Timp1 mRNA expression levels in each group. **(C–E)** WB analysis of the protein expression levels in each group. **(F)** ROS contents in each group detected by the DCFH-DA assay. The data are presented as the means ± SEMs of three replicates. ^∗^*p* < 0.05, versus the CON group; ^#^*p* < 0.05, versus the NOX4^GV 358Lv^ group.

Upon elucidation of the important roles of NOX4 and RhoA in a series of behaviors during HSC activation, we further evaluated the relationships of their protein expression with the two signaling pathways to determine the regulatory relationship between NOX4 and RhoA and investigate the inhibitory functions of UA toward these pathways. After TGF-β1 stimulation, the NOX4 mRNA expression in the CON+UA and NOX4i groups was decreased compared to that in the CON group (*p* < 0.05), and the NOX4 mRNA expression in the NOX4^GV 358Lv^+UA group was decreased compared to that in the NOX4^GV 358Lv^ group (*p* < 0.05) (Figure 4A). The mRNA expression levels of Rhoa, Rock1, Alphasma, Col1a1, and Timp1 in the CON+UA, NOX4i, and RhoAi groups were decreased compared to those in the CON group (*p* < 0.05) (Figure 4A,B), and the mRNA levels of these genes in the NOX4^GV 358Lv^+UA group were decreased compared to those in the NOX4^GV 358Lv^ group (*p* < 0.05) (Figure 4A,B). Compared to that in the CON group, the Mmp1 mRNA expression in the CON+UA, NOX4i, and RhoAi groups was increased (*p* < 0.05), and the Mmp1 mRNA expression in the NOX4^GV 358Lv^+UA group was increased compared to that in the NOX4^GV 358Lv^ group (*p* < 0.05) (Figure 4B). These results indicated that inhibition of Nox4 mRNA expression inhibits the expression of Rhoa mRNA, but inhibition of Rhoa mRNA does not inhibit the expression of Nox4 mRNA. Moreover, these findings demonstrated that inhibition of Nox4 and Rhoa mRNA expression inhibits the expression of Rock1, Alphasma, Col1a1, and Timp1 mRNA but promotes the expression of Mmp1 mRNA. Furthermore, these results showed that UA inhibits the expression of Nox4, Rhoa, Rock1, Alphasma, Col1a1, and Timp1 mRNA but promotes the expression of Mmp1 mRNA.

### UA Reduces the Protein Expression of RhoA/ROCK1 Signaling Pathways Through NOX4 in HSCs

The expression levels of proteins in the NOX4/ROS and RhoA/ROCK1 pathways were investigated (Figure 4C). The NOX4 protein expression in the CON+UA and NOX4i groups was decreased compared to that in the CON group (*p* < 0.05), and it was lower in the NOX4^GV 358Lv^+UA group than in the NOX4^GV 358Lv^ group (*p* < 0.05) (Figure 4D). The expression levels of RhoA, ROCK1, α-SMA, Collagen-I, and TIMP1 in the CON+UA were decreased in the NOX4i and RhoAi groups compared to those in the control group. Also, these proteins show the same trends in the NOX4^GV 358Lv^+UA group when compared with those in NOX4^GV 358Lv^ group (*p* < 0.05) (Figure 4D,E). Compared to that in the CON group, the protein expression of MMP1 in the CON+UA, NOX4i, and RhoAi groups was increased (*p* < 0.05), while MMP1 expression was decreased in the NOX4^GV 358Lv^ group compared to that in the control group (*p* < 0.05). Additionally, the MMP1 protein expression in the NOX4^GV 358Lv^+UA group was increased compared to that in the NOX4^GV 358Lv^ group (*p* < 0.05) (Figure 4E). These results suggested that inhibition of NOX4 protein expression inhibits RhoA protein expression, but inhibition of RhoA protein expression does not inhibit NOX4 protein expression. These findings also showed that inhibition of the NOX4 and RhoA protein expression levels inhibits RhoA, ROCK1, α-SMA, Collagen-I, and TIMP1 protein expression but promotes the protein expression of MMP1. Furthermore, these data indicated that UA inhibits the expression of NOX4, RhoA, ROCK1, α-SMA, Collagen-I, and TIMP1 but promotes the expression of MMP1. Additionally, the expression of MMP2 and MMP9 was not significantly different among all these groups, and treatment of the NOX4i and RhoAi groups with UA did not result in further reduction of the α-SMA expression level (Supplementary Figure 2).

### UA Suppresses the ROS Level in HSCs *via* NOX4 and Not RhoA

To further explore the mechanism of NOX4, RhoA, and UA in the regulation of cellular activity, the intracellular ROS level was evaluated. The content of ROS in the CON+UA and NOX4i groups was lower than that in the CON group (*p* < 0.01). The ROS level in the NOX4^GV 358Lv^ group was significantly higher than that in the CON group (*p* < 0.01), and the ROS level in the NOX4^GV 358Lv^+UA group was lower than that in the NOX4^GV 358Lv^ group (*p* < 0.01) (Figure 4F). These results showed that NOX4 promotes the ROS content in HSCs and that RhoA does not affect the ROS content. Moreover, these findings demonstrated that UA reduces the ROS content in HSCs by inhibiting NOX4 but not RhoA, which confirmed the results of our preliminary studies.

### HSC Behaviors Change After NOX4^GV 358Lv^-RhoAi Double Transduction

**FIGURE 5:**
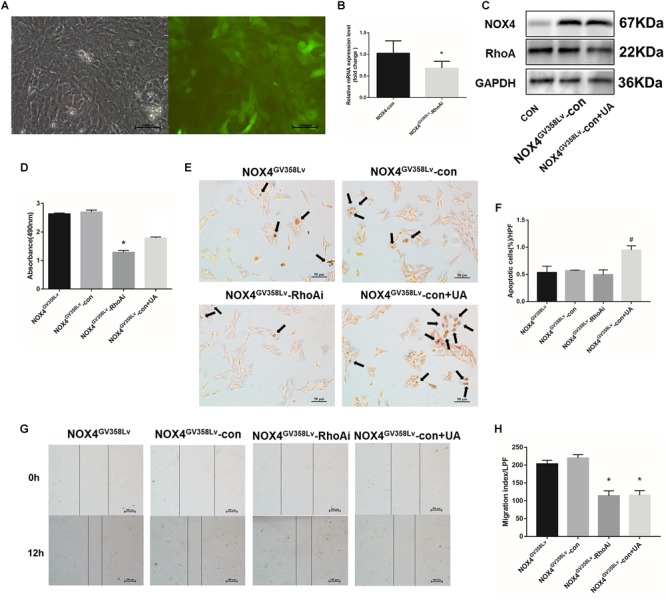
Construction and identification of NOX4^GV 358Lv^-RhoA shRNA stable cell lines. **(A)** The NOX4^GV 358Lv^ cell line was transfected with a lentivirus containing RhoA shRNA. Cells were observed under an optical microscope (left) and a fluorescence microscope (right) in the same field of vision. Original magnification, 200×; **(B)** RT-qPCR analysis of Nox4 mRNA in the NOX4 shRNA stable cell line. **(C)** WB analysis of the NOX4 protein levels in the NOX4^GV 358Lv^-RhoAi group. **(D)** Cell proliferation detected by the MTS assay. **(E,F)** Cell apoptosis levels detected by the TUNEL assay. Black arrows: typical apoptotic cells. Percentage of apoptotic cells = apoptotic cells/cell count per high-power field; 10 different fields of vision were randomly selected from each group. Original magnification, 200×; **(G,H)** Cell scratch assay. Original magnification, 100×. The data are presented as the means ± SEMs of three replicates. ^∗^*p* < 0.05, versus the NOX4^GV 358Lv^-con group; ^#^*p* < 0.05, versus the NOX4^GV 358Lv^-RhoAi group.

To further clarify the regulatory relationship between NOX4 and RhoA as well as the target of UA intervention, lentiviral particles containing RhoA shRNA were transduced into NOX4^GV 358Lv^ stable HSCs, which were stably passaged. The transduction effects are shown in Figure 5A–C. The proliferation of the NOX4^GV 358Lv^-RhoAi group was lower than that of the NOX4^GV 358Lv^-con group (lentivirus with a scramble oligonucleotide sequence transduced into the NOX4^GV 358Lv^ stable cell line) (*p* < 0.05), but there was no significant difference in the proliferation of the NOX4^GV 358Lv^-con+UA, NOX4^GV 358Lv^-con, and NOX4^GV 358Lv^-RhoAi groups (Figure 5D). The proportion of apoptotic cells in the NOX4^GV 358Lv^-con+UA group was significantly higher than that in the NOX4^GV 358Lv^-con group (*p* < 0.05), but the proportion of apoptotic cells in the NOX4^GV 358Lv^-RhoAi group was not significantly different from that in the NOX4^GV 358Lv^-con group (Figure 5E,F).

**FIGURE 6:**
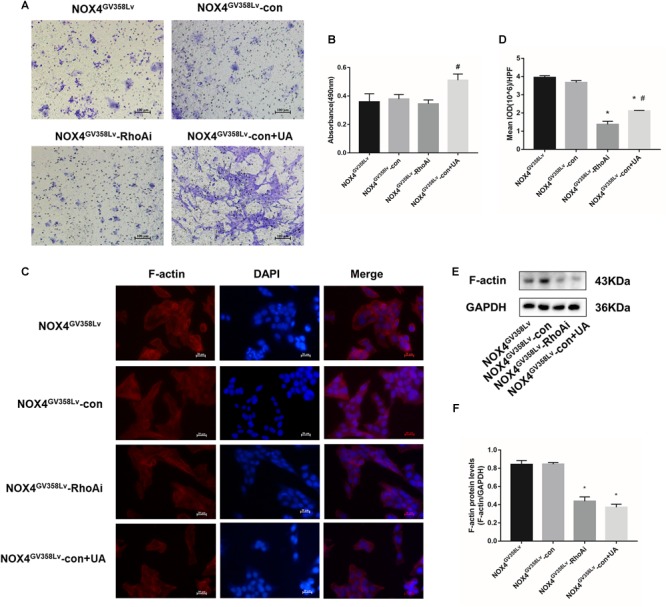
The invasion levels and F-actin polymerization in the NOX4^GV 358Lv^-RhoA shRNA stable cell line. **(A,B)** Cell invasion detected by the Transwell invasion system. Original magnification, 200×. **(C,D)** F-actin immunofluorescence staining in each group. Mean IOD values were measured by Image Pro Plus 6.0 software; 10 different fields of visions were selected. Original magnification, 400×. **(E,F)** WB analysis of F-actin in each group. The data are presented as the means ± SEMs of three replicates. ^∗^*p* < 0.05, versus the NOX4^GV 358Lv^-con group; ^#^*p* < 0.05, versus the NOX4^GV 358Lv^-RhoAi group.

In the cell scratch assay, the migration indexes of the NOX4^GV 358Lv^-RhoAi and NOX4^GV 358Lv^-con+UA groups were much lower than that of the NOX4^GV 358Lv^-con group (*p* < 0.05) (Figure 5G,H). The degree of invasion of the NOX4^GV 358Lv^-con+UA group was higher than that of the NOX4^GV 358Lv^-RhoAi group (*p* < 0.05), but no statistically significant difference was observed in the invasion degrees of the NOX4^GV 358Lv^-RhoAi and NOX4^GV 358Lv^-con groups (Figure 6A,B). These findings indicated that RhoA promotes the proliferation, migration, and invasion of HSCs, but the mechanism of HSC apoptosis is independent of RhoA, and UA still has an inhibitory effect on NOX4 in these processes.

### Cytoskeleton F-Actin Polymerization Attenuates in NOX4^GV 358Lv^-RhoAi HSCs

To further investigate the impact of RhoAi on behavioral changes in the HSC cell line, cytoskeleton F-actin staining was used. The NOX4^GV 358Lv^-RhoAi and NOX4^GV 358Lv^-con+UA groups had lower F-actin staining intensity and protein content than the NOX4^GV 358Lv^-con group (*p* < 0.05) (Figure 6C,D). WB analysis of F-actin showed the same tendency, but the F-actin level in the NOX4^GV 358Lv^-RhoAi group was not significantly different from that in the NOX4^GV 358Lv^-con+UA group (Figure 6E,F). Collectively, these results confirmed that RhoA has positive effects on F-actin polymerization, and NOX4 seems to be a regulatory protein toward RhoA.

### NOX4/ROS Is a Positive Regulator of the RhoA/ROCK1 Pathway

**FIGURE 7:**
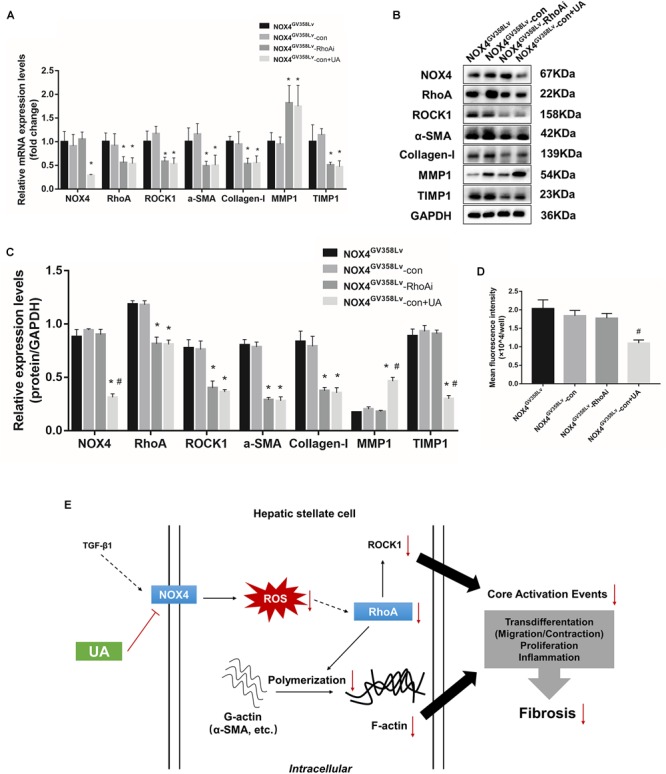
mRNA and protein expression changes in the NOX4^GV 358Lv^-RhoA shRNA stable cell line and direct binding analysis of NOX4 and RhoA. **(A)** RT-qPCR analysis of the mRNA expression levels in each group. **(B,C)** WB analysis of the protein expression levels in each group. **(D)** ROS contents in each group detected by the DCFH-DA assay. The data are presented as the means ± SEMs of three replicates. ^∗^*p* < 0.05, versus the CON group; ^#^*p* < 0.05, versus the NOX4^GV 358Lv^ group. **(E)** Recapitulative overview scheme of the study.

NOX4- and RhoA-related mRNA and protein levels were evaluated. The NOX4 mRNA and protein expression levels in the NOX4^GV 358Lv^-con+UA group were decreased compared to those in the NOX4^GV 358Lv^-con group (*p* < 0.05) but not compared to those in the NOX4^GV 358Lv^-RhoAi group (Figure 7A,C,D). Compared to those in the NOX4^GV 358Lv^-con group, the mRNA expression levels of Rhoa, Rock1, Alphasma, and Col1a1 in the NOX4^GV 358Lv^-RhoAi group were decreased (*p* < 0.05) (Figure 7A), and the same tendency was observed for the RhoA, ROCK1, α-SMA, and Collagen-I protein expression levels (Figure 7B,C). The MMP1 and TIMP1 protein levels in the NOX4^GV 358Lv^-con+UA group were significantly different from those in the NOX4^GV 358Lv^-con group (both *p* < 0.05) but not from those in the NOX4^GV 358Lv^-RhoAi group (Figure 7B,C).

Next, the ROS contents in each group were detected to confirm whether ROS are affected by RhoA interruption. The intracellular ROS content in the NOX4^GV 358Lv^-con+UA group was lower than that in the NOX4^GV 358Lv^-RhoAi group (*p* < 0.05). Moreover, there was no significant difference in the ROS levels between the NOX4^GV 358Lv^-RhoAi and NOX4^GV 358Lv^-con groups (Figure 7D). These results indicated that RhoA might have a positive effect on HSC activation, and NOX4/ROS is a positive regulator of the RhoA/ROCK pathway. Additionally, the above results confirmed that UA has the inhibitory impact of reducing the expression levels of NOX4 and RhoA/ROCK1 and well as those of fibrosis-related proteins, such as α-SMA, Collagen-I, MMP-1, and TIMP1.

### Interaction Between NOX4 and RhoA Proteins

Finally, we wondered whether a direct interaction exists between NOX4 and RhoA such that NOX4 acts as an upstream molecule regulating the RhoA/ROCK1 pathway, which seems to be essential for fibrosis regulation. For this purpose, NOX4^GV 358Lv^ HSCs were lysed and analyzed for coimmunoprecipitation with different proteins (p67^phox^ and Rac1; NOX4 and Rac1; p67^phox^ and RhoA; and NOX4 and RhoA) (Supplementary Figure 3A) to verify the presence of binding between different protein combinations. The p67^phox^ and Rac1 proteins were bound to each other, but no binding was detected between the following protein pairs: NOX4 and Rac1, p67^phox^ and RhoA, and NOX4 and RhoA.

The Biacore protein interaction analysis system was utilized to further explore the direct binding possibilities between the NOX4 and RhoA proteins. The NOX4 monoclonal antibody was conjugated to a CM5 chip, and the full-length human recombinant NOX4 protein was bound to the antibody. RhoA was formulated at different concentrations (0, 22.5, 45, 90, 180, and 360 μg/mL). The ligand solution flowed uniformly across the surface of the chip, and the *in vitro* combination of the full-length NOX4 protein with the purified RhoA protein did not exhibit a typical concentration-dependent binding curve (Supplementary Figure 3B). These results indicated that there might be no direct interaction between the NOX4 and RhoA proteins.

## Discussion

Chronic liver injury caused by different etiologies (e.g., viral infections, alcohol abuse, nonalcoholic steatohepatitis, and cholestasis) can lead to excessive accumulation of ECM proteins (e.g., type I collagen), resulting in hepatic fibrosis and eventual progression to fibrosis (Singh et al., 2017). MFBs activated during liver injury are a critical source of ECM proteins and can promote the development and progress of hepatic fibrosis (Chen et al., 2018).

The activation of HSCs into MFBs is a central event in the pathogenesis of fibrosis (Oh et al., 2016). Evidence has suggested that NOX is a multicomponent complex that catalyzes the conversion of oxygen molecules to ROS and plays an essential role in the activation of HSCs (Gan et al., 2018). Studies have shown that NOX isomers, including NOX1, NOX2, and NOX4, as well as ROS produced by multiple NOX-derived proteins are involved in the regulation of HSC activation (Lan et al., 2015). NOX4 mediates the signal transduction of TGF-β1 and other major hepatic fibrogenic factors, which can lead to HSC activation and hepatocyte apoptosis (Jiang et al., 2012).

Rho GTPase is a crucial molecule in many cellular signal transduction pathways, and RhoA, Rac1, and Cdc42 are essential members (Boyle et al., 2018). Studies have shown that RhoA and its downstream signaling molecules are expressed in hepatic vascular smooth muscle cells, vascular endothelial cells, and HSCs, increasing hepatic vascular resistance and aggravating liver fibrosis (Hennenberg et al., 2006; Trebicka et al., 2007). Recent studies have found that RhoA participates in the regulation of hepatic fibrosis by regulating the migration, adhesion, contraction, proliferation, and apoptosis of HSCs (Li et al., 2012). However, the relationship between RhoA/ROCK1 and NOX4/ROS has not elucidated.

Our previous results showed that UA is an inhibitor of NOXs on the membrane of liver cells (including liver parenchymal cells and nonparenchymal cells), which can significantly inhibit HSC activation and reverse liver fibrosis. Therefore, we hope to further elucidate the molecular mechanism by which UA modifies HSC activation. In this study, we found that both UA and Fasudil could improve liver fibrosis in WT mice, which was consistent with the conclusions of our previous research and with those of other research groups. Interestingly, *in vivo* experiments on NOX4^-/-^ mice showed that the liver fibrosis induced by CCl_4_ was significantly improved compared with that in WT CCl_4_ mice, and neither Fasudil nor UA could further improve the liver fibrosis of NOX4^-/-^ mice. Therefore, we hypothesized that NOX4 knockout may also influence the action site (RhoA/ROCK1 pathway) of Fasudil, as the effect was not apparent in NOX4^-/-^ fibrotic mice.

In this study, lentivirus transduction was utilized to interrupt the expression of NOX4 and RhoA in HSCs, and compared with those in the control group, the levels of proliferation, migration, and cytoskeleton aggregation in the NOX4i group were significantly reduced. In contrast, upon NOX4 overexpression, the proliferation and migration levels of the NOX4^GV 358Lv^ group were significantly increased compared to those in the control group, as expected. Thus, both NOX4 and RhoA clearly play essential roles in the activation of HSCs, and their functional activation can significantly promote the proliferation, migration, and other cytoskeletal movements of HSCs. More importantly, upon inhibiting NOX4 expression, under the stimulus of TGF-β1, RhoA and its essential effect on the downstream molecule ROCK1 as well as the fibrosis-associated Alphasma, Col1a1, Mmp1, and Timp1 mRNA and their protein expression levels were significantly decreased. However, the NOX4 expression level was not affected when RhoA expression was inhibited (RhoAi group), and the ROS level in the RhoAi group was not significantly different from that in the control group. Other proteins related to fibrosis progression, such as MMP2 and MMP9, did not explain much of the variation among the groups, potentially due to the isolated system of stimulated HSCs, which had no interactions with Kupffer cells, liver sinusoidal endothelial cells (LSECs) or leukocytes, and also may correlated with our experiment system.

Above all, NOX4/ROS is a regulatory pathway of RhoA/ROCK1 and plays an essential regulatory role in the proliferation, migration, and activation of HSCs. These results were confirmed by the application of UA to the NOX4 overexpression group (NOX4^GV 358Lv^) and the NOX4^GV358Lv^-RhoAi group; fibrosis-related proliferation, migration, and cytoskeleton polymerization were significantly inhibited, and the mRNA and protein expression levels of related molecules were also significantly decreased. While UA can substantially promote the apoptosis of HSCs, this effect is unrelated to the two targets of NOX4 and RhoA. We will not discuss this phenomenon in detail in this manuscript.

It is also worth noting that the ability to migrate and invade is not unique to tumor cells. Various types of cells, including HSCs [such as osteoblasts (Cao et al., 2018), astrocytes (Zhang et al., 2018), macrophages (Kammrath Betancor et al., 2018), neutrophils (Sadik et al., 2018), vascular endothelial cells (Li et al., 2015), and trophoblast cells (Wang et al., 2015)], have been demonstrated to have the ability to migrate and/or invade. HSCs are located within Disse’s space, which is approximately 0.4 μm wide and located between intrahepatic sinusoidal endothelial cells and hepatocytes (Shang et al., 2018). Disse’s space, which is closely attached to LSECs and hepatocytes, is irregular in shape, as it has a round or irregular shaped cell body and several apical cells protruding around the hepatic sinusoids (Poonkhum et al., 2015). The basement membrane-like matrix in Disse’s space provides a tightly regulated microenvironment. Disse’s space allows macromolecules to better pass from the sinusoids to hepatocytes and maintains the integrity and function of liver cells (Poonkhum et al., 2015). Studies have shown that many activated HSCs accumulate in sites of intrahepatic inflammation. Thus, the activation of HSCs in these spaces is an important sign of liver fibrosis progression; however, this progression is also indicated by other more typical markers of HSC activation, such as α-SMA, Collagen-I, and other fibrosis-related proteins (Puche et al., 2013). However, it cannot be concluded that these behaviors are most likely related to cytoskeletal regulation. Therefore, we used the Transwell invasion system to explore the general ability of HSC motion transfer and collagen formation to simulate the complex behavioral changes of HSCs *in vivo*.

The invasion level in the RhoAi group was lower than that in the NOX4i group, and similar results were found for the NOX4^GV 358Lv^-RhoAi group, which was unexpected. Matrigel, a type of 3D matrix, is a type of ECM extracted from mouse tumors that consists of laminin, type IV collagen, heparan sulfate proteoglycan (HSPG), and nestin as well as some growth factors. Importantly, MMP secretion is negatively correlated with the degree of fibrosis, and cell migration is related to cytoskeletal deformability (Puche et al., 2013). RhoA is a downstream signaling molecule of NOX4 during the activation of HSCs but is closely related to cytoskeleton activity. Inhibition of RhoA expression significantly increases the cytoskeletal vasodilation capacity, although the upstream NOX4/ROS pathway is activated. Because the expression of RhoA was inhibited and the cytoskeletal contraction was not successfully controlled, some cells could not pass through the small pores in the Transwell chambers.

Finally, we wondered whether NOX4 and RhoA directly bind with any other proteins during the execution of these regulatory functions. Earlier studies reported that the Rho GTPase family homologous protein Rac1 could directly bind to the NOX2-activated subunit p67^phox^ (Diekmann et al., 1994) and directly participate in NOX2 activation. We suggested that a direct binding relationship exists between NOX4 and RhoA, which plays a role of mutual regulation. However, no direct connection was detected between NOX4 and RhoA or between p67^phox^ and RhoA in either the CoIP experiment or in the Biacore protein interaction analysis system, and further studies and analyses of NOX4 and other activated subunits are required.

Therefore, we believe that NOX4/ROS is the upstream regulatory pathway of RhoA/ROCK1. Based on the above findings, we believe that UA can further inhibit RhoA/ROCK1 expression *via* NOX4/ROS and suppress HSC activation (Figure 7E) and even hepatic fibrosis *in vivo* through this manner; however, more experiments should be performed to confirm these findings.

## Ethics Statement

This study was carried out in accordance with the recommendations of “humane care in compliance with institutional guidelines, Institutional Animal Care and Use Committee of the First Affiliated Hospital of Nanchang University.” The protocol was approved by the “Institutional Animal Care and Use Committee of the First Affiliated Hospital of Nanchang University.”

## Author Contributions

CH is responsible for the experiments and article writing. DG is responsible for the project design. FL and SW are responsible for the animal experiments. FL is responsible for the molecular biology experiments. JC is responsible for the cell slide and color rendering, grading, etc. AW and BL are responsible for assisting the data processing and picture modification. XZ is responsible for the final modification of the manuscript.

## Conflict of Interest Statement

The authors declare that the research was conducted in the absence of any commercial or financial relationships that could be construed as a potential conflict of interest.

## Abbreviations

ANOVA: analysis of variance
DAPI: 4′,6-diamidino-2-phenylindole dihydrochloride
EDC: 1-ethyl-3-(3′-dimethylaminopropyl)carbodiimide
GAPDH: glyceraldehyde-3-phosphate dehydrogenase
GFP: green fluorescent protein
HSC: hepatic stellate cell
MFB: myofibroblast
MMP: matrix metalloproteinase
NHS: *N*-hydroxysuccinimide
NOX: NADPH oxidase
RhoA: ras homolog family member A
ROCK1: Rho-associated kinase 1
ROS: reactive oxygen species
RT-qPCR: reverse transcription-quantitative polymerase chain reaction
SEM: standard error of mean
α-SMA: smooth muscle α-actin
TGF-β1: transforming growth factor-β1
TIMP-1: tissue inhibitor of metalloproteinase-1
TUNEL: TdT-mediated dUTP nick-end labeling
UA: ursolic acid
WB: western blot

## References

[B1] Aoyama T. Paik Y. H. Watanabe S. Laleu B. Gaggini F. Fioraso-Cartier L. (2012). Nicotinamide adenine dinucleotide phosphate oxidase in experimental liver fibrosis: GKT137831 as a novel potential therapeutic agent. *Hepatology* 56 2316–2327. 10.1002/hep.25938 22806357 PMC3493679

[B2] Boyle S. T. Kular J. Nobis M. Ruszkiewicz A. Timpson P. Samuel M. S. (2018). Acute compressive stress activates RHO/ROCK-mediated cellular processes. *Small GTPases* 10.1080/21541248.2017.1413496 29455593 PMC7549670

[B3] Cao R. Shao J. Hu Y. Wang L. Li Z. Sun G. (2018). microRNA-338-3p inhibits proliferation, migration, invasion, and EMT in osteosarcoma cells by targeting activator of 90 kDa heat shock protein ATPase homolog 1. *Cancer Cell Int.* 18:49. 10.1186/s12935-018-0551-x 29618948 PMC5879792

[B4] Chen W. Zhang Z. Yao Z. Wang L. Zhang F. Shao J. (2018). Activation of autophagy is required for Oroxylin A to alleviate carbon tetrachloride-induced liver fibrosis and hepatic stellate cell activation. *Int. Immunopharmacol.* 56 148–155. 10.1016/j.intimp.2018.01.029 29414645

[B5] Crosas-Molist E. Fabregat I. (2015). Role of NADPH oxidases in the redox biology of liver fibrosis. *Redox Biol.* 6 106–111. 10.1016/j.redox.2015.07.005 26204504 PMC4804101

[B6] Diekmann D. Abo A. Johnston C. Segal A. W. Hall A. (1994). Interaction of Rac with p67phox and regulation of phagocytic NADPH oxidase activity. *Science* 265 531–533. 8036496 10.1126/science.8036496

[B7] Gan D. Zhang W. Huang C. Chen J. He W. Wang A. (2018). Ursolic acid ameliorates CCl4-induced liver fibrosis through the NOXs/ROS pathway. *J. Cell. Physiol.* 233 6799–6813. 10.1002/jcp.26541 29672850 PMC6055678

[B8] He W. Shi F. Zhou Z. W. Li B. Zhang K. Zhang X. (2015). A bioinformatic and mechanistic study elicits the antifibrotic effect of ursolic acid through the attenuation of oxidative stress with the involvement of ERK, PI3K/Akt, and p38 MAPK signaling pathways in human hepatic stellate cells and rat liver. *Drug Des. Dev. Ther.* 9 3989–4104. 10.2147/dddt.s85426 26347199 PMC4529259

[B9] Hennenberg M. Biecker E. Trebicka J. Jochem K. Zhou Q. Schmidt M. (2006). Defective RhoA/Rho-kinase signaling contributes to vascular hypocontractility and vasodilation in cirrhotic rats. *Gastroenterology* 130 838–854. 10.1053/j.gastro.2005.11.029 16530523

[B10] Jiang J. X. Chen X. Serizawa N. Szyndralewiez C. Page P. Schroder K. (2012). Liver fibrosis and hepatocyte apoptosis are attenuated by GKT137831, a novel NOX4/NOX1 inhibitor in vivo. *Free Radic. Biol. Med.* 53 289–296. 10.1016/j.freeradbiomed.2012.05.007 22618020 PMC3392471

[B11] Kammrath Betancor P. Hildebrand A. Bohringer D. Emmerich F. Schlunck G. Reinhard T. (2018). Activation of human macrophages by human corneal allogen in vitro. *PLoS One* 13:e0194855. 10.1371/journal.pone.0194855 29617399 PMC5884541

[B12] Klein S. Rick J. Lehmann J. Schierwagen R. Schierwagen I. G. Verbeke L. (2017). Janus-kinase-2 relates directly to portal hypertension and to complications in rodent and human cirrhosis. *Gut* 66 145–155. 10.1136/gutjnl-2015-309600 26385087

[B13] Lan T. Kisseleva T. Brenner D. A. (2015). Deficiency of NOX1 or NOX4 prevents liver inflammation and fibrosis in mice through inhibition of hepatic stellate cell activation. *PLoS One* 10:e0129743. 10.1371/journal.pone.0129743 26222337 PMC4519306

[B14] Lee Y. A. Wallace M. C. Friedman S. L. (2015). Pathobiology of liver fibrosis: a translational success story. *Gut* 64 830–841. 10.1136/gutjnl-2014-306842 25681399 PMC4477794

[B15] Li F. Yuan Y. Guo Y. Liu N. Jing D. Wang H. (2015). Pulsed magnetic field accelerate proliferation and migration of cardiac microvascular endothelial cells. *Bioelectromagnetics* 36 1–9. 10.1002/bem.21875 25338938

[B16] Li L. Wang J. Y. Yang C. Q. Jiang W. (2012). Effect of RhoA on transforming growth factor beta1-induced rat hepatic stellate cell migration. *Liver Int.* 32 1093–1102. 10.1111/j.1478-3231.2012.02809.x 22498718

[B17] Manickam N. Patel M. Griendling K. K. Gorin Y. Barnes J. L. (2014). RhoA/Rho kinase mediates TGF-β(1)-induced kidney myofibroblast activation through Poldip2/Nox4-derived reactive oxygen species. *Am. J. Physiol. Renal Physiol.* 307 F159–F171. 10.1152/ajprenal.00546.2013 24872317 PMC4101629

[B18] Meng Y. Li T. Zhou G. S. Chen Y. Yu C. H. Pang M. X. (2015). The angiotensin-converting enzyme 2/angiotensin (1-7)/Mas axis protects against lung fibroblast migration and lung fibrosis by inhibiting the NOX4-derived ROS-mediated RhoA/Rho kinase pathway. *Antioxid. Redox Signal.* 22 241–258. 10.1089/ars.2013.5818 25089563 PMC4283064

[B19] Nakamura M. Verboon J. M. Parkhurst S. M. (2017). Prepatterning by RhoGEFs governs Rho GTPase spatiotemporal dynamics during wound repair. *J. Cell Biol.* 216 3959–3969. 10.1083/jcb.201704145 28923977 PMC5716286

[B20] Ni J. Dong Z. Han W. Kondrikov D. Su Y. (2013). The role of RhoA and cytoskeleton in myofibroblast transformation in hyperoxic lung fibrosis. *Free Radic. Biol. Med.* 61 26–39. 10.1016/j.freeradbiomed.2013.03.012 23517783 PMC3849210

[B21] Nomikou E. Livitsanou M. Stournaras C. Kardassis D. (2018). Transcriptional and post-transcriptional regulation of the genes encoding the small GTPases RhoA, RhoB, and RhoC: implications for the pathogenesis of human diseases. *Cell. Mol. Life Sci.* 75 2111–2124. 10.1007/s00018-018-2787-y 29500478 PMC11105751

[B22] Oh Y. Park O. Swierczewska M. Hamilton J. P. Park J. S. Kim T. H. (2016). Systemic PEGylated TRAIL treatment ameliorates liver cirrhosis in rats by eliminating activated hepatic stellate cells. *Hepatology* 64 209–223. 10.1002/hep.28432 26710118 PMC4917440

[B23] Paik Y. H. Iwaisako K. Seki E. Inokuchi S. Schnabl B. Osterreicher C. H. (2011). The nicotinamide adenine dinucleotide phosphate oxidase (NOX) homologues NOX1 and NOX2/gp91(phox) mediate hepatic fibrosis in mice. *Hepatology* 53 1730–1741. 10.1002/hep.24281 21384410 PMC3082608

[B24] Paik Y.-H. Kim J. Aoyama T. De Minicis S. Bataller R. Brenner D. A. (2014). Role of NADPH oxidases in liver fibrosis. *Antioxid. Redox Signal.* 20 2854–2872. 10.1089/ars.2013.5619 24040957 PMC4026397

[B25] Poonkhum R. Showpittapornchai U. Pradidarcheep W. (2015). Collagen arrangement in space of Disse correlates with fluid flow in normal and cirrhotic rat livers. *Microsc. Res. Tech.* 78 187–193. 10.1002/jemt.22460 25536906

[B26] Puche J. E. Saiman Y. Friedman S. L. (2013). Hepatic stellate cells and liver fibrosis. *Compr. Physiol.* 3 1473–1492. 10.1002/cphy.c120035 24265236

[B27] Sadik C. D. Miyabe Y. Sezin T. Luster A. D. (2018). The critical role of C5a as an initiator of neutrophil-mediated autoimmune inflammation of the joint and skin. *Semin. Immunol.* 37 21–29. 10.1016/j.smim.2018.03.002 29602515

[B28] Schuppan D. Surabattula R. Wang X. Y. (2018). Determinants of fibrosis progression and regression in NASH. *J. Hepatol.* 68 238–250. 10.1016/j.jhep.2017.11.012 29154966

[B29] Shang L. Hosseini M. Liu X. Kisseleva T. Brenner D. A. (2018). Human hepatic stellate cell isolation and characterization. *J. Gastroenterol.* 53 6–17. 10.1007/s00535-017-1404-4 29094206

[B30] Singh S. Muir A. J. Dieterich D. T. Falck-Ytter Y. T. (2017). American gastroenterological association institute technical review on the role of elastography in chronic liver diseases. *Gastroenterology* 152 1544–1577. 10.1053/j.gastro.2017.03.016 28442120

[B31] Sirokmany G. Donko A. Geiszt M. (2016). Nox/Duox family of NADPH oxidases: lessons from knockout mouse models. *Trends Pharmacol. Sci.* 37 318–327. 10.1016/j.tips.2016.01.006 26861575

[B32] Tacke F. (2017). Targeting hepatic macrophages to treat liver diseases. *J. Hepatol.* 66 1300–1312. 10.1016/j.jhep.2017.02.026 28267621

[B33] Trebicka J. Hennenberg M. Laleman W. Shelest N. Biecker E. Schepke M. (2007). Atorvastatin lowers portal pressure in cirrhotic rats by inhibition of RhoA/Rho-kinase and activation of endothelial nitric oxide synthase. *Hepatology* 46 242–253. 10.1002/hep.21673 17596891

[B34] Wang Y. Y. Zhou R. Zhou B. Wang T. Zhang L. Luo D. (2015). Overexpression of heparanase is associated with preeclampsia by inhibiting invasion of trophocytes. *Int. J. Clin. Exp. Med.* 8 18107–18114. 26770407 PMC4694307

[B35] Yu S. S. Chen B. Huang C. K. Zhou J. J. Huang X. Wang A. J. (2017). Ursolic acid suppresses TGF-beta1-induced quiescent HSC activation and transformation by inhibiting NADPH oxidase expression and Hedgehog signaling. *Exp. Ther. Med.* 14 3577–3582. 10.3892/etm.2017.5001 29042951 PMC5639307

[B36] Zhang F. Huang Y. Wang B. Zhong C. Liu X. Ding S. (2018). LINC00673 silencing inhibits cell migration and invasion by suppressing PI3K/AKT signaling in glioma. *Neuroreport* 29 718–722. 10.1097/wnr.0000000000001022 29621055

