# Supplementary material for: Interaction Mechanisms Between the NOX4/ROS and RhoA/ROCK1 Signaling Pathways as New Anti- fibrosis Targets of Ursolic Acid in Hepatic Stellate Cells
Source: Front Pharmacol. 2019 May 3;10:431. doi: 10.3389/fphar.2019.00431 (PMC6510285; doi:10.3389/fphar.2019.00431)
Supplement: Supplementary file 1 [file Data_Sheet_1.pdf]

Supplementary Figures

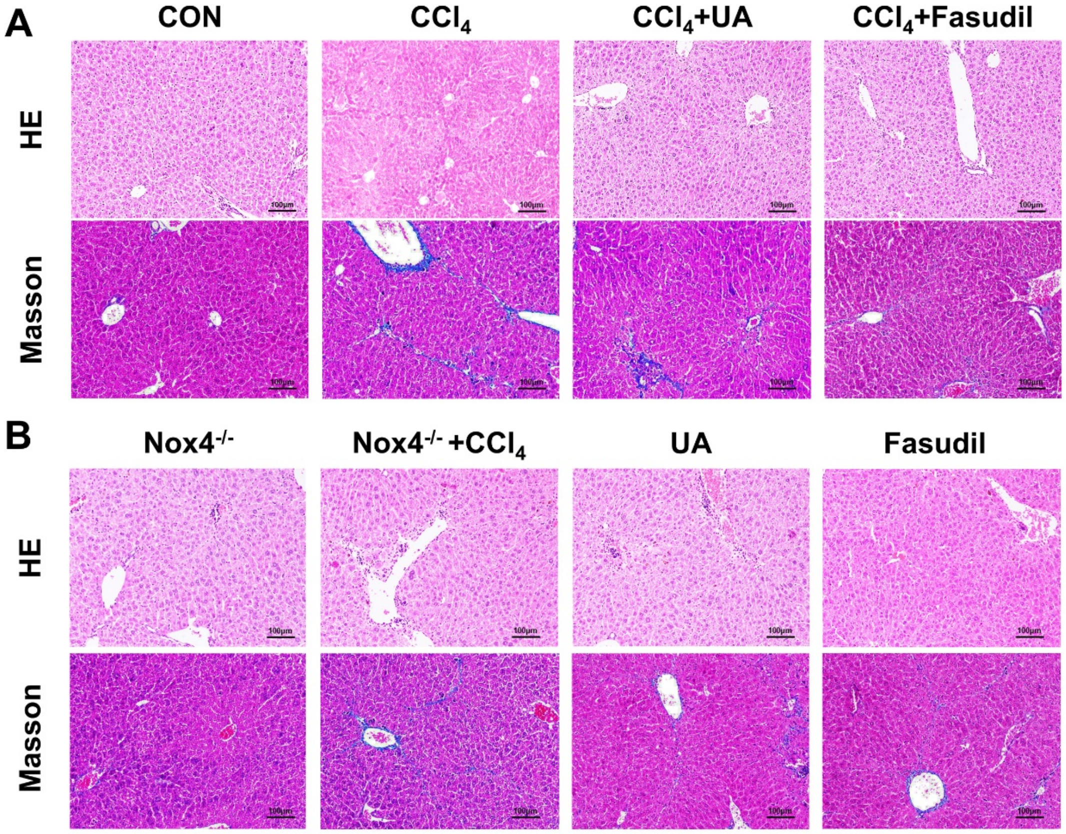

Supplementary Figure 1. (A). HE & Masson staining of liver tissues from each group of wild-type mice. (B). HE & Masson staining of tissues from each group of NOX4$^{-/-}$ mice. Original magnification, ×100.

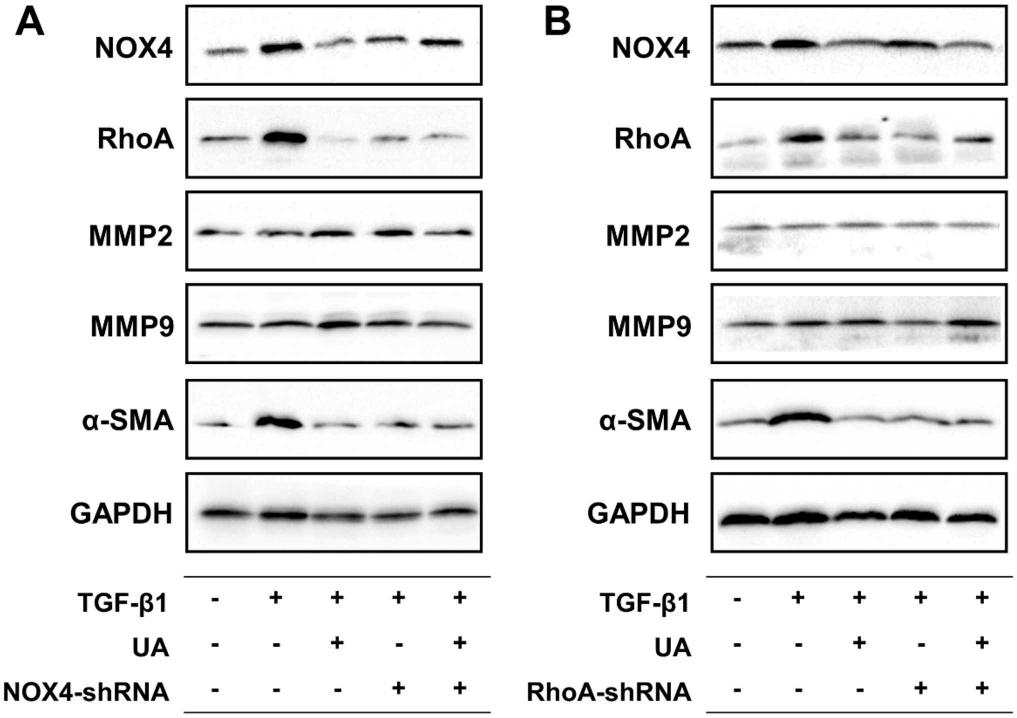

Supplementary Figure 2. (A). Western blot analysis of NOX4-shRNA with or without UA intervention under the stimulation of TGF-β1-induced HSC activation. (B). Western blot analysis of NOX4-shRNA with or without UA intervention under the stimulus of TGF-β1-induced HSC activation.

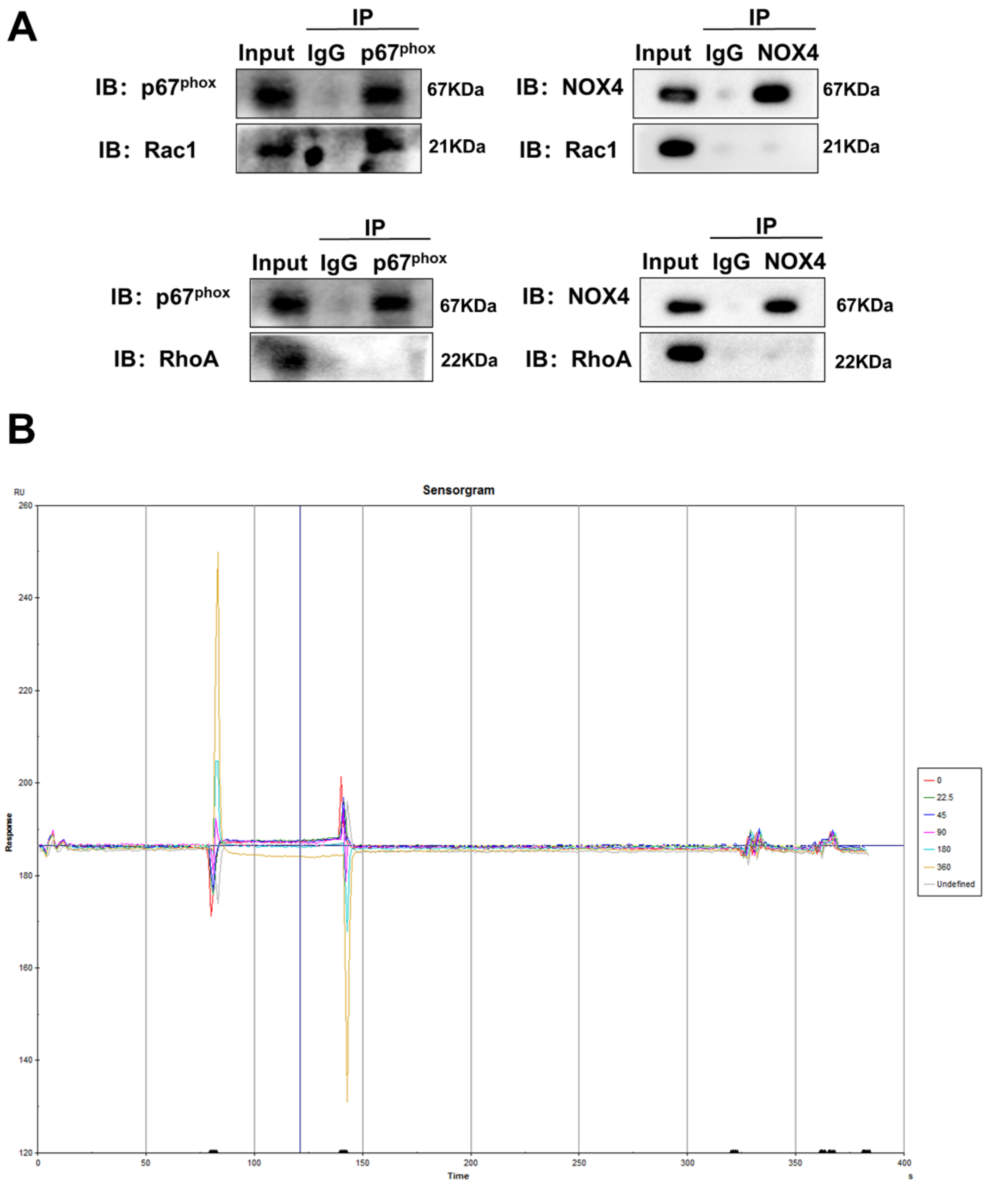

Supplementary Figure 3. (A). Coimmunoprecipitation analysis of different proteins. (B). Binding dissociation curve between the NOX4 and RhoA proteins generated using the Biacore protein interaction analysis system. The anti-NOX4 antibody was bound to the CM5 chip, and the full-length recombinant NOX4 protein was bound to the antibody. RhoA was diluted to 0 μg/mL, 22.5 μg/mL, 45 μg/mL, 90 μg/mL, 180 μg/mL, 360 μg/mL with a buffer and loaded to observe its binding activity.
